# Effects of gelatin and oxytocin supplementation in a long-term semen extender on boar semen quality and fertility potential

**DOI:** 10.5713/ab.23.0233

**Published:** 2023-08-30

**Authors:** Vibuntita Chankitisakul, Nalinee Tubtimtong, Wuttigrai Boonkum, Thevin Vongpralub

**Affiliations:** 1 Department of Animal Science, Faculty of Agriculture, Khon Kaen University, Khon Kaen, 40002, Thailand; 2 Network Center for Animal Breeding and Omics Research, Khon Kaen University, Khon Kaen, 40002, Thailand

**Keywords:** Boar, Cold Semen, Conception Rate, Gelatin, Litter Size, Solid Storage

## Abstract

**Objective:**

This study investigated the efficacy of different concentrations of gelatin supplementation in long-term semen extender on boar semen quality during storage for 10 days at 17°C. Additionally, oxytocin was added to stored semen to enhance fertility.

**Methods:**

In Experiment 1, boar semen was collected, diluted with gelatin at concentrations between 0% and 2.5% (w/v) and mixed with a semen extender. Then, it was kept in a refrigerator at 17°C and stored for 10 days. In Experiment 2, the sperm quality was examined after adding 0, 5, and 10 IU oxytocin per artificial insemination dose to the most effective semen extender from Experiment 1 and placing it in a refrigerator at 17°C for 10 days. In Experiment 3, the fertility potential in terms of non-return rate and litter size was determined using the most effective solid-stored semen supplemented with oxytocin.

**Results:**

The results indicated that sperm quality decreased with increasing storage time (p<0.05). The sperm quality in terms of total motility, progressive motility, and viable sperm with intact acrosomes and high mitochondrial potential was the highest with 1.5% gelatin supplementation (p<0.001) on all days of storage. Treatment with oxytocin did not affect sperm quality (p>0.05). The non-return rate and litter size after insemination with semen supplemented with 1.5% gelatin and 10 IU of oxytocin after 8 to 10 days of storage were comparable to those of the control group (p>0.05).

**Conclusion:**

A semen extender as a solid medium supplemented with 1.5% gelatin successfully preserved boar semen for a long storage duration. Treatment with oxytocin did not affect sperm quality. In addition, the fertility capacity using 1.5% gelatin with 10 IU oxytocin and stored for 8 to 10 days was acceptable and comparable to that of short-term storage.

## INTRODUCTION

Currently, artificial insemination (AI) with 50 to 100 mL of diluted semen by either cervical or post cervical deposition with a total sperm dose between 1.0 and 2.5×10^9^ is routinely practiced in the pig industry [1]. The extended semen is preserved at 17°C and stored for several days, depending on the type of preservation medium. If the AI is carried out on the day of semen collection or after semen dilution, using a short-term extender is appropriate for AI on the farm. Moreover, in cases that require prolonging the preservation time, i.e., smallholder farms in rural areas, a long-term extender is generally appropriate. This latter type of extender extends the lifetime of boar semen by acting as an energy source for sperm metabolism, facilitating pH buffering for sperm cell waste and providing ions for membrane and cell balance. However, its limited ability to function as a storage medium without reducing the fertilizing capacity of semen is a concern [2]; this limitation is probably related to oxidative stress, which affects the survival of sperm during cooled storage.

When sperm is stored in the liquid state, the sperm cells, microorganisms, and other particles are deposited at the bottom as sediment. Sedimentation mainly decreases pH and increases the toxic metabolic products in sedimented regions [3]. Gently rotating to facilitate resuspension has been recommended to increase the lifespan of semen. In contrast, some research has reported the detrimental effect of rotation on semen preservation [4,5]. However, the underlying mechanisms remain to be elucidated. Therefore, to prevent sedimentation, the supplementation of gelatin in semen extenders is an interesting alternative method that has been reported to be successful in preserving the semen of many species, such as rabbits [3,6], sheep [7] and chickens [8]. Gelatin has properties as a collagen hydrolysate, which is a large molecule that does not enter the spermatozoa cell. The viscosity of the medium is increased during storage in a temperature range below 20°C; thus, the motility is diminished [9]. In addition, sedimentation is prevented in the solid state. Moreover, it was reported that gelatin supplementation in rabbit semen extenders allows extending the storage duration [6]. This is an interesting issue regarding whether we can prolong the storage duration while still maintaining acceptable sperm fertility. However, to the best of our knowledge, the application of gelatin supplementation in semen extenders during cooled storage is limited to pig semen. Corcini et al [10] reported that semen stored in a gelatin-supplemented short-term extender had satisfactory quality for up to 96 h. However, the semen was used to inseminate after only 2 h of storage.

In addition to better sperm quality, uterine conditions at the insemination time are also necessary for pig fertility. During natural breeding, increasing uterine contraction induced by oxytocin positively affected sperm transportation into the oviduct [11]. However, the level of oxytocin in sows was reported to decrease sharply due to the lack of physical contact between boars and sows during AI [12], while the concentration of oxytocin in the seminal plasma of boars was found to be independent of this factor, instead differing among different boar species and the ejaculates of boars of the same species [13]. Therefore, measuring the oxytocin concentration in seminal plasma is recommended to determine a better selection of boars to successfully improve AI. However, it is not routinely practical to evaluate every ejaculation. Some reports have proposed using oxytocin as a semen additive in the extender before insemination to improve sperm fertility in terms of farrowing rates and litter sizes [14,15]. This is an easily applicable, effective method for increasing sperm fertility potential. However, the previous studies described that oxytocin was supplemented to the semen dose approximately 15 min before the first insemination [14,15]; meanwhile, the present study was designed by adding the oxytocin on top of the semen extender since preparing the semen dose and storing for up to 10 days. The addition of oxytocin to semen extender and storing for a longer duration has never been reported before; thus, before insemination by using the semen supplemented with gelatin and oxytocin, it is necessary to confirm whether those have no detrimental effect on stored sperm.

Accordingly, the present study aimed to prolong the semen cold storage time by supplementing a long-term semen extender with different concentrations of gelatin between 0% and 2.5% (w/v) for a conventional storage temperature of 17°C and to study the effects of this supplementation on the parameters of sperm quality. Additionally, the detrimental effect of oxytocin supplementation (5 and 10 IU) in solid cold-stored semen was determined, as the previous report suggested that seminal plasma oxytocin concentrations were found to be highly related to *in vivo* fertility [14]. The fertility in terms of non-return rate and litter size was examined using a solid long-term semen extender supplemented with oxytocin.

## MATERIALS AND METHODS

This study was conducted under animal care approved by the Institutional Animal Care and Use Committee based on the Ethics of Animal Experimentation of the National Research Council of Thailand (record no. AEKKU 32/2558). All chemicals utilized in this study were purchased from Sigma Aldrich Chemical (St. Louis, MO, USA) unless otherwise specified.

### Animals

Six boars (BA81 breed, a commercial species modified by Betagro Agro Industry Co. Ltd., Kranuan, Khon Kaen, Thailand) with proven fertility were raised in individual pens in an evaporative housing system. The ages of the boars ranged from 2 to 3 years old. Fresh water was provided *ad libitum*, while feed was given twice a day in accordance with the requirements of boars for semen collection [16]. Semen was routinely collected from the boars once to twice a week for AI.

Twenty-three weaned crossbred sows (parity 1–6) were used for AI. After weaning, they were housed in individual pens in an open housing system for estrus detection, breeding, and gestation. Fresh water was provided *ad libitum*, while feed was given twice a day in accordance with the requirements of sows [16].

### Semen collection

Ejaculated semen as bulk samples [17] were collected from each boar using a gloved-hand technique. The semen samples were transferred to the laboratory within 30 minutes after collection. Fresh sperm quality was determined. The semen volume was measured by a digital weight scale. The pH value was assessed using pH indicator paper. The motility of sperm was determined under a light microscope at 400× magnification. The sperm concentration was measured by a hemocytometer. Only ejaculates with ≥70% motility were selected for further procedures.

### Semen extender and gelatin preparation

Semen samples were extended in the Butschwiler extender according to Weitze [18] with minor modifications. It was composed of 35 g d-glucose, 6.9 g sodium citrate, 2.25 g ethylenediaminetetraacetic acid (EDTA), 1.0 g sodium bicarbonate, 1 g polyvinyl alcohol (PVA), 5.65 g trizma base, 3.15 g citric acid, 0.05 g L-cysteine, 150 mg lincomycin, and 300 mg spectinomycin in 1,000 mL double distilled water. The pH was 7.2, and the osmotic pressure was 284 g/100 mL.

The gelatin extender was prepared by adding type A gelatin (G1890) at different concentrations (according to Experiment 1) to the Butschwiler extender. After preparation, the gelatin extender was in a liquid state when the environment temperature was at room temperature (at 25°C to 28°C), while it became the solid stage when the gelatin extender was kept below 20°C.

### Experimental design

*Experiment 1: to examine the optimal level of gelatin supplementation for sperm quality*: After collection and evaluation, the semen was divided into five aliquots to dilute with the semen extender containing different concentrations of gelatin to a final concentration of 3×10^7^ sperm/mL. Different concentrations of gelatin at 1.0%, 1.5%, 2.0%, and 2.5% based on previous reports in rabbit sperm [3,6], sheep sperm [7], and rooster sperm [8] were added to the Butschwiler extender. Butschwiler extender without gelatin supplementation served as a control. The diluted semen was placed in a refrigerator at 17°C and stored for 10 days. Semen quality was evaluated in terms of sperm motility, viable sperm with intact acrosomes and high mitochondrial potential, and the pH values after storage at Days 0 (after the cooling process), 4, 7, and 10. The experiment was repeated 8 times.

*Experiment 2: to examine the detrimental effect of oxytocin supplementation in the semen extender with regard to sperm quality*: According to the results of Experiment 1, a 1.5% gelatin group was selected to combine with oxytocin and determine whether oxytocin added in solid cold-stored semen for the long term would have a detrimental effect on sperm quality in this experiment. Oxytocin (General Drugs Houses. Co., Ltd, Pathumthani, Thailand) at 0, 5, and 10 IU per AI dose based on previous reports [10,15] was added on top of the Butschwiler extender supplemented with 1.5% gelatin after storage for 1 h (allowing the gelatin extender to become solid). The diluted semen was placed in a refrigerator at 17 °C and stored for 10 days. Semen quality was evaluated in terms of sperm motility, viable sperm with intact acrosomes and high mitochondrial potential, and the pH values after storage at Days 0 (after the cooling process), 4, 7, and 10. The experiment was repeated 8 times.

*Experiment 3: to examine the non-return rate and litter size*: According to the results of Experiment 2, Butschwiler extender supplemented with 1.5% gelatin and 10 IU of oxytocin was selected to determine the non-return rate and litter size in this experiment. For this purpose, AI was carried out in 23 sows (control, 11; treatment, 12). The sows were randomized and assigned to be inseminated with either i) semen diluted with Butschwiler extender after 1 to 3 days of storage at 17°C or ii) semen diluted with Butschwiler extender supplemented with 1.5% gelatin and 10 IU of oxytocin after 8 to 10 days of storage at 17°C. Before insemination, the stored semen was warmed at 37°C for 30 min and homogenized by gentle shaking. Pregnancy was confirmed by estrus detection. The sows not returning to estrus in the next estrus cycle were considered conceptus. The number of total born piglets was considered as the litter sizes at farrowing.

### Semen evaluation

*Sperm motility*: Total motility and progressive motility were determined using a computer-assisted semen analyzer, CASA (Hamilton Thorne Biosciences, version 12 TOX VIOS, Beverly, MA, USA), as described by Ratchamak et al [19]. A minimum of 300 sperm per sample were counted. Motility was expressed as the percentage of motile sperm cells. Total motility was expressed as the percentage of sperm making any sort of movement. Progressive motility was expressed by sperm that swam in a mostly straight line.

*Sperm viability, acrosome integrity, and mitochondrial function*: The percentage sperm viability, acrosome integrity, and mitochondrial potential were determined by fluorescent multiple staining using propidium iodide (Live/dead sperm viability kit L7011; Invitrogen, Waltham, MA, USA), fluorescein isothiocyanate-labeled peanut (Arachishypogaea), agglutinin (FITC-PNA), and 5, 5’, 6, 6’-tetrachloro-1, 1’, 3, 3’-tetraethylbenzimidazolylcarbocyanine iodide (JC-1) staining, respectively, as previously described by Ratchamak et al [19]. Three hundred sperm were assessed immediately by a fluorescence microscope with a triple filter, showing a set of UV-2E/C (excitation 340 to 380 nm and emission 435 to 485 nm), B-25/C (excitation 465 to 495 nm and emission 515 to 555 nm) and G-2E/C (excitation 540 to 525 nm and emission 605 to 655 nm) at ×400 magnification. Viable sperm with intact acrosomes and high mitochondrial potential was confirmed by observing colorless sperm heads and plasma membranes with red–orange JC-1 in the midpiece region of sperm [20].

*pH*: The pH value was assessed using pH indicator paper. The pH paper was immersed in a semen sample and removed quickly. After a few seconds, the color change was observed on the pH indicator paper, and the pH reading was recorded.

*Artificial insemination*: Estrus detection was performed using boar–sow contact together with the back-pressure test twice a day after weaning. Only weaned sows that returned to estrus within 7 days were used in this study. The sows were inseminated with 80 mL of diluted semen (3×10^9^ sperm cells) two times at 12 h and 24 h after standing heat detection using the conventional method.

### Statistical analysis

Experiments 1 and 2 were analyzed using analysis of variance with a split-plot fitted into a completely randomized design with 8 replications. The factors (treatment, days of storage, and interaction between treatment and days of storage) were compared for differences using Duncan’s new multiple-range test. The overall differences between factor means were considered significant when p<0.05. Experiment 3 was analyzed using a Group t-test to determine the significant differences between the two experimental groups.

Data were analyzed using SAS 9.0 statistical software (SAS Institute, Inc., Cary, NC, USA). All data were first tested for normality and homogeneity of variance and then analyzed by the Proc general linear model procedure for Experiments 1 and 2 and by the Proc t-test procedure for Experiment 3.

## RESULTS

### Experiment 1: to examine the optimal level of gelatin supplementation for sperm quality

Table 1 shows that the interaction effect between treatment and days of storage was highly significant in terms of total motility, progressive motility, and viable sperm with intact acrosomes and high mitochondrial potential (p<0.001). However, the differences in pH values were not significant (p>0.05).

The sperm quality in terms of total motility, progressive motility, and the proportion of viable sperm with intact acrosomes and high mitochondrial potential was highest with 1.5% gelatin supplementation (p<0.001) on all days of storage. However, the sperm quality continued to decrease with increasing storage time (p<0.001) regardless of treatment. Minor pH values decreased after longer storage, but these differences were insignificant (p>0.05). The highest gelatin dose of 2.5% negatively affected sperm parameters, resulting in the lowest progressive motility after 4 days of storage (p< 0.001).

### Experiment 2: to examine the detrimental effect of oxytocin supplementation in the semen extender with regard to sperm quality

The effects of oxytocin supplementation in boar semen extender with 1.5% gelatin are presented in Table 2. Only days of storage had a significant effect on sperm quality (p<0.05), which decreased when storage time increased regardless of treatment. In contrast, treatment with oxytocin did not affect sperm quality (p>0.05). Therefore, the extender supplemented with 1.5% gelatin and 10 IU of oxytocin was used to determine the fertility potential in Experiment 3.

### Experiment 3: to examine the non-return rate and litter size

The non-return rate and litter size after insemination with semen supplemented with 1.5% gelatin and 10 IU of oxytocin after 8 to 10 days of storage were comparable to those of the control group, as shown in Table 3 (p>0.05).

## DISCUSSION

In the present study, gelatin supplementation in a long-term extender with oxytocin was first examined to extend the storage time for up to 10 days while maintaining an effective fertility potential. The results indicate that 1.5% gelatin supplementation in the semen extender medium was the most effective for improving sperm quality during storage. Moreover, higher gelatin supplementation at 2% negatively affected sperm quality. Treatment with oxytocin did not affect sperm quality. The non-return rate and litter size using the solid cold-stored semen for up to 8–10 days were acceptable compared to the control’s.

When the semen was stored in the liquid state, the sperm cells, microorganisms, and other particles were deposited at the bottom as sediment. The sedimentation of sperm cells during liquid storage decreases the pH and increases the toxic metabolic products in the sedimented regions [3]. Increasing the viscosity of dilute semen to become temporarily solidified can reduce sperm metabolic demand [3,6]. The positive effect on sperm quality of gelatin supplementation at 1.5% concentration by preventing sedimentation suggests the possibility of maintaining semen for a longer period, resulting in improved quality of boar semen when preserving longer for up to 10 days. This is an advantage when transporting diluted semen in cases that require prolonging the preservation time, i.e., smallholder farms in rural areas. However, a significant negative effect of high gelatin dosage on sperm quality, especially progressive motility, was noted after 4 days of storage in the present study. Moreover, the detrimental effect of 3% gelatin supplementation was not observed in a previous study [10], which might be related to their evaluation after short-term storage (within 72 h). Esbenshade and Nebel [21] speculated that with a higher viscosity, sperm use more energy for motility, subsequently resulting in energy loss during movement [11,21]. Our study suggests that high levels of gelatin are not recommended for supplementation in the long-term solid preservation of boar semen.

It is reported that pH has an influence on sperm physiology [22]. Numerous extracellular stimuli may affect the intracellular pH of the samples; consequently, the storage medium can tremendously affect diluted sperm. In the present study, the pH variations among groups with different concentrations of gelatin supplementation during storage for up to 10 days were minimized and not significantly different among groups and storage times, even though the storage time was extended (Table 1). This might be explained by the fact that gelatin prevents sedimentation; thus, sperm metabolism is reduced. However, it is supposed that the pH of semen stored in a liquid state (control group) should fluctuate due to the buildup of the metabolic products of sperm when storage time increases. Interestingly, the differences in pH were not different compared to those of other factors. A possible explanation for this might be related to the Butschwiler extender used in our study. The Butschwiler extender is suitable for long-term storage by acting as an energy source for sperm metabolism, facilitating pH buffering for sperm cell waste, and providing ions for membrane and cell balance. The Butschwiler extender contains EDTA, a potential chelating agent that has a significant function in blocking the action of calcium as a mediator of sperm capacitation and the acrosome reaction during cold storage [23]. Additionally, it uses bicarbonate as a buffer system, which is quite an efficient buffer for preventing pH fluctuation, as reported by Purdy et al [24].

In addition, the Butschwiler extender was modified by replacing bovine serum albumin (BSA) with PVA in the present study. Since the components of BSA are not defined, its high price likely prohibits the commercial use of some extenders. Furthermore, BSA is an animal-derived component that must be avoided. Both Cheng [25] and Zhou et al [26] suggested that PVA can be used as a substitute for BSA in long-term semen extenders. Similarly, BSA can be replaced with PVA for the liquid storage of buck semen [27]. The mechanism by which PVA protects spermatozoa during semen storage is unknown. However, it might be speculated that PVA has a superior ability to maintain sperm survival during preservation. In addition, antibiotics were added to the modified Butschwiler extender in the present study, which might imply effective antimicrobial activity. Therefore, based on the abovementioned findings, we inferred that the modified Butschwiler extender in the present study facilitated pH buffering for sperm cell waste and provided ions for membrane and cell balance.

Supplementing semen extender with oxytocin as an additive has been proposed to improve sperm fertility in terms of farrowing rates and litter sizes by enhancing sperm transportation from the deposition site to the oviduct [28], especially during seasonal infertility in the summer season [15,16]. However, in previous studies, the semen dose was supplemented with oxytocin approximately 15 min prior to the first insemination. Meanwhile, this study was designed by adding oxytocin on top of the semen extender since preparing the semen dose since Day 0 and storing it for up to 10 days, presumably easy and practical for use by local farmers. Therefore, we must confirm whether oxytocin added to the semen extender and stored for long-term storage had no detrimental effect on sperm as in Experiment 2. Table 2 revealed that only days of storage had a significant effect on sperm quality which decreased when storage time increased. In contrast, treatment with oxytocin did not affect sperm quality. Therefore, inseminating the semen diluted with 1.5% gelatin and oxytocin, which is stored for up to 10 days, is possible. For the determination of oxytocin dose in Experiment 3, we considered that the results of oxytocin concentrations between 5 and 10 IU were not different (Table 2; p>0.05). Therefore, we chose oxytocin at 10 IU, a higher concentration of oxytocin than that in previous reports (4 to 5 IU), to test the fertility potential in Experiment 3 since seminal plasma oxytocin concentrations were found to be highly related to *in vivo* fertility [14]. In other words, the best farrowing rates in sows have ejaculates with the highest seminal plasma oxytocin concentrations that positively activate the myometrial contractions facilitating sperm transportation to the utero-tubal junction and increasing the fertilizing capacity [28].

Interestingly, the progressive motility of the sperm in the long-term semen extender significantly decreased by less than 20% on Day 10 of storage; however, the fertility of sows inseminated with semen with oxytocin after long-term solid storage for 8 to 10 days was comparable to that of the control sows (Table 3). Sperm motility is an important parameter for identifying sperm quality. However, there is evidence that sperm motility is not correlated with fertility when stored boar semen is used. In contrast, the number of inseminated sperm per dose appears to be more influential [29,30]. A semen extender generally dilutes boar semen at 2 to 3×10^9^ sperm cells per dose, while a minimum dose of 1.5×10^9^ is acceptable for commercial AI services [29]. Tardif et al [30] demonstrated that a suboptimal dose of 0.3×10^9^ sperm cells was comparable to a dose of 3×10^9^ sperm cells in terms of the non-return rate. Therefore, it might be inferred that even the progressive motility is lower than that of the control. However, in the present study, a sperm concentration of 3×10^9^ sperm cells per dose was used. The motile sperm numbers of the long-term storage group were sufficient without any deficiency for deposition at the sperm reservoirs and fertilization with matured oocytes after ovulation.

Furthermore, litter size is influenced by acrosomal intact sperm [29]. The comparable litter size in the present study confirmed the effectiveness of viable sperm with intact acrosomes and high mitochondrial potential in the long-term storage group, which was higher than 75% after long-term storage (Table 2). Despite the lower sperm progressive motility, the viable sperm with intact acrosomes and high mitochondrial potential was obviously still greater, which was also similarly reported by Bielas et al [31]. In conclusion, it appears that sperm viability had a positive effect on fertilization.

## CONCLUSION

In conclusion, this is the first report on a long-term boar semen extender as a solid cold-stored semen supplemented with oxytocin. The 1.5% gelatin supplementation level in the semen extender medium was the most effective for improving sperm quality after storage. Treatment with oxytocin did not affect sperm quality. The non-return rate and litter size using the solid cold-stored semen supplemented with 10 IU oxytocin and stored at temperature of 17°C for 8 to 10 days were acceptable and comparable to that of short-term storage.

## Figures and Tables

**Table 1:** Percentage of total motility, progressive motility, and viable sperm with intact acrosomes and high mitochondrial potential of semen extender supplemented with gelatin at various concentrations after 0, 4, 7, and 10 days of a conventional storage temperature of 17°C

Days of storage (d)	Treatment (% concentration of gelatin)					SEM	p-value		
	
	0%	1.0%	1.5%	2.0%	2.5%		Treatment	Days of storage	Interaction
Total motile sperm (%)									
0	82.36^b,w^	81.99^b,w^	86.44^a,w^	80.26^b,w^	80.48^b,w^	0.88	<0.0001	<0.0001	<0.0001
4	63.34^c,x^	64.93^c,x^	74.30^a,x^	72.30^a,x^	70.37^b,x^	1.67			
7	54.80^b,y^	57.89^b,y^	61.32^a,y^	57.63^b,y^	48.83^c,yz^	1.65			
10	32.20^d,z^	33.72^d,z^	49.83^a,z^	42.05^b,z^	40.40^b,c^	2.50			
Progressive motile sperm (%)									
0	52.76^c,w^	57.57^b,w^	66.54^a,w^	65.13^a,w^	57.95^b,w^	2.03	<0.0001	<0.0001	<0.0001
4	40.08^c,x^	43.49^b,x^	48.30^a,x^	42.61^b,x^	38.79^c,x^	1.30			
7	27.40^b,y^	30.71^a,y^	31.47^a,y^	25.33^b,y^	20.04^c,y^	1.63			
10	19.55^b,z^	20.16^b,z^	29.88^a,y^	20.43^b,z^	13.49^c,z^	2.08			
Live normal sperm (%)									
0	81.86^a,w^	84.37^a,w^	83.03^a,w^	82.03^a,w^	81.48^a,w^	0.41	<0.0001	<0.0001	<0.0001
4	73.57^b,x^	81.78^a,x^	81.77^a,x^	79.73^a,x^	76.48^ab,x^	1.27			
7	72.15^b,x^	77.99^a,y^	79.44^a,x^	78.51^ab,x^	74.74^ab,y^	1.07			
10	66.81^b,y^	73.71^ab,z^	77.58^a,y^	72.96^ab,y^	71.39^ab,z^	1.38			
pH									
0	7.53	7.51	7.46	7.45	7.45	0.01	0.2056	0.3045	0.3211
4	7.45	7.42	7.38	7.36	7.37	0.01			
7	7.29	7.28	7.26	7.25	7.24	0.01			
10	7.25	7.26	7.25	7.24	7.23	0.00			

SEM, standard error of the mean.

a–d Means within a row with superscripts indicate significant differences (p<0.05).

w–z Means within a column with superscripts indicate significant differences (p<0.05).

**Table 3:** Conception rate and litter size using a long-term extender supplemented with 1.5% gelatin and 10 IU of oxytocin

Treatments^1)^	Sow number (n)	Non-return rate (%)	Litter size (n)
Control	11	100.00	11.81±2.04
Long-term extender	12	91.67	11.00±2.68
p-value	-	0.1644	0.4501

1) Control, semen diluted with Butschwiler extender after 1 to 3 days of storage; Long-term extender, semen diluted with Butschwiler extender supplemented with 1.5% gelatin and 10 IU of oxytocin after 8 to 10 days of storage.

**Table 2:** Percentage of total motility, progressive motility, and sperm viability with intact acrosomes and high mitochondrial potential of semen extender supplemented with gelatin at various concentrations after 1, 4, 7, and 10 days of a conventional storage temperature of 17°C

Days of storage (d)	Treatment (% concentration of oxytocin)			SEM	p-value		
	
	0 IU	5 IU	10 IU		Treatment	Days of storage	Interaction
Total motility (%)							
1	78.56^a^	81.85^a^	85.63^a^	2.81	0.3158	0.0439	0.3506
4	75.42^a^	78.33^a^	78.41^b^	2.99			
7	53.60^b^	59.67^b^	58.31^c^	2.56			
10	40.96^c^	44.29^c^	41.09^d^	3.03			
Progressive motility (%)							
1	54.93^a^	58.42^a^	55.74^a^	2.87	0.2277	0.0414	0.4511
4	40.25^b^	42.27^b^	45.36^b^	3.09			
7	24.39^c^	26.70^c^	28.28^c^	3.05			
10	17.10^d^	19.31^d^	16.73^d^	1.80			
Sperm viability with intact acrosomes and high mitochondrial potential (%)							
1	88.59^a^	88.46^a^	86.78^a^	0.96	0.2682	0.0066	0.0927
4	84.26^b^	84.74^b^	84.26^b^	1.14			
7	82.81^b^	83.67^b^	82.39^b^	1.47			
10	75.16^c^	75.18^c^	74.85^c^	2.17			

SEM, standard error of the mean.

a–d Means within a column with superscripts indicate significant differences (p<0.05).
